# Adrenergic Contraction of Human Prostate Tissues Is Maintained Under the Rab GTPase Inhibitors Nexhinib20 and CID1067700

**DOI:** 10.1002/prp2.70331

**Published:** 2026-10-16

**Authors:** Luca Troffer, Peibin Cen, Michael Brandstetter, Yajie Xu, Patrick Keller, Leo Stadelmeier, Philipp Weinhold, Alexander Tamalunas, Christian G. Stief, Martin Hennenberg

**Affiliations:** ^1^ Department of Urology LMU University Hospital, LMU Munich Munich Germany

**Keywords:** α_1_‐adrenoceptor, α_1_‐blocker, benign prostatic hyperplasia (BPH), lower urinary tract symptoms (LUTS), posttranslational regulation, Rab27, Rab7, voiding symptoms

## Abstract

α_1_‐Adrenoceptors are an important drug target in benign prostatic hyperplasia (BPH) and have been intensively studied at the expression level, but their posttranslational regulation in the prostate is widely unknown. Here we examined modulation of prostate smooth muscle contractions by inhibitors for RabGTPases, which are involved in intracellular transport of internalized receptors and in vesicle function. Tissues were obtained from laser enucleation for BPH and from radical prostatectomy without prior BPH surgery. Contractions were induced in an organ bath, with and without the Rab27 inhibitor nexhinib20 or the Rab7 inhibitor CID1067700. Nexhinib20 reduced contractions induced by electric field stimulation (EFS) at 10 μM in laser‐enucleated tissues (*E*_max_ 79% [34–125] of KCl‐induced contraction in controls, 40% [6–74] of KCl with nexhinib20, *p* = 0.0234), but not at 1 μM or in prostatectomized tissues. Contractions by phenylephrine, methoxamine and noradrenaline were resistant to consistent modulation by nexhinib20 and CID1067700, both at 1 μM and 10 μM and in both types of prostate tissues. EFS‐induced contractions of laser‐enucleated and prostatectomized tissues remained unaltered with CID1067700. The lack of consistent effects of CID1067700 despite previously reported Rab7‐dependent regulation of α_1A_‐adrenoceptors questions the transferability of findings from receptor overexpression systems and biochemical small GTPase assays to endogenous receptor regulation in human tissues. Divergent responsiveness of neurogenic and agonist‐induced contractions to nexhinib20 and CID1067700 suggests Rab7‐independent regulation of α_1_‐adrenoceptor‐mediated contraction, but a possible presynaptic role of Rab27 in neurotransmission of human prostate smooth muscle.

## 1 Introduction

Alpha_1_‐adrenoceptors induce contraction of prostate smooth muscle and are a central target in medical treatment of voiding symptoms suggestive of benign prostatic hyperplasia (BPH) [1, 2]. Voiding symptoms in BPH result from bladder outlet obstruction, caused by increased prostate smooth muscle tone, by prostate growth or both [3]. Alpha_1_‐adrenoceptor antagonists (α_1_‐blockers) are the first line option for their medical treatment, and improve symptoms presumably by an inhibition of α_1_‐adrenergic prostate smooth muscle contraction [1, 2, 3]. BPH and voiding symptoms belong to the most common benign diseases in elderly men [3]. However, although α_1_‐blockers are among the most commonly prescribed drug classes in men aged ≥ 70 years [4], regulation and function of α_1_‐adrenoceptors in the prostate remain surprisingly poorly understood [2], particularly in comparison to adrenoceptors in other organs. Despite their widespread use, the efficacy of α_1_‐blockers is limited [2], so that advanced understanding of their function may help to develop future options to optimize the efficacy of drug treatment in BPH.

Previous research addressing regulation of prostate α_1_‐adrenoceptors focussed on their expression levels and on the subtype‐selectivity of antagonists. Typically, G protein‐coupled receptors are also subject to profound posttranslational regulation, including desensitization, internalization, and either recycling or degradation of internalized receptors. Posttranslational regulation of adrenoceptors has been intensively studied for cardiac β‐adrenoceptors, but also exemplarily for non‐prostatic α_1_‐adrenoceptors. The current knowledge on posttranslational regulation of prostatic adrenoceptors is highly limited, in particular in human tissues [2]. Stromal cells in the human prostate express β‐arrestin‐2 and clathrins, both interacting with endogenous α_1A_‐adrenoceptors and suggesting a posttranslational regulation [5, 6]. However, functional implications or the involvement of further mechanisms of posttranslational regulation are completely unknown for α_1_‐adrenoceptors of the human prostate.

Following internalization, posttranslationally regulated receptors are located to endosomal vesicles. The intracellular transport of vesicles and their further processing is largely regulated by Rab GTPases. In cell culture models of non‐prostatic cells with stable receptor overexpression, internalized α_1A_‐adrenoceptors are first located to early endosomes, which is paralleled by interaction with Rab7 [7]. Typically, the interaction of adrenoceptors with Rab7 results in further transport to late endosomes and finally in receptor degradation [8]. While the interaction of internalized receptors with Rab7 has been shown for overexpressed, non‐prostatic α_1A_‐adrenoceptors [7], implications for endogenous α_1A_ in the human prostate are yet unknown. In addition, intracellular vesicle transport is essential in neurotransmission, including the release of noradrenaline from synaptic vesicles to postsynaptic α_1_‐adrenoceptors on prostate smooth muscle cells in neurogenic contractions in prostate tissues. Exocytotic processes again involve Rab GTPases, including Rab27 among others [9, 10].

Based on evidence for Rab7‐mediated trafficking of internalized adrenoceptors in cell culture models with receptor overexpression, and because endogenous noradrenaline is a contractile neurotransmitter in the prostate, effects of small molecule inhibitors for Rab7, Rab27 or further Rab isoforms could be expected. To date, only some few inhibitors with claimed specificity to RabGTPases are available, which do not cover the whole range of all Rab GTPases that are potentially involved in adrenoceptor regulation. None of them has been explored in smooth muscle contraction. Here, we examined effects of the Rab7 inhibitor CID1067700 and of the Rab27 inhibitor nexinhib20 on contractions of human prostate tissues from laser‐enucleation for BPH, and from patients without prior surgery for BPH.

## 2 Methods

### 2.1 Human Prostate Tissues

Human prostate tissues were collected from holmium and thulium laser enucleation of the prostate (HoLEP, ThuLEP) performed for BPH, and from patients undergoing radical prostatectomy (rPx) for prostate cancer but without previous surgery for BPH. Tissues from both cohorts were examined and analyzed separately. These cohorts represent different clinical settings rather than formally defined stages of BPH, but nevertheless differ considerably in their BPH‐specific clinical characteristics. Patients needing laser enucleation of the prostate are characterized by advanced, medication‐refractory voiding symptoms, high postvoid residual urine volume (PVR) or complications, while the majority of patients undergoing rPx but without prior surgery for BPH show only mild or moderate symptoms [11]. Specifically, laser‐enucleation for BPH is performed in patients with advanced BPH, that is, with medication‐refractory symptoms, high PVR, and/or with imminent or experienced complications [1]. In large cohorts, the IPSS of patients undergoing laser‐enucleation at our department ranges from 17 to 21, the *Q*_max_ from 7.6 to 11 mL/s, the PVR from 60 to 100 mL, and the prostate volume from 90 to 95 mL [12, 13, 14]. Mean age is 71 years [12, 13, 14], and 31.6%–38% of these patients are catheterized for urinary retention before the surgery [14, 15]. In contrast, BPH may be present in 80% of patients undergoing rPx for prostate cancer, but none of the patients included here was treated by surgery for BPH prior to our study. The median IPSS in prostatectomized prostate cancer patients at our department averages out at 7 [16], and ranges < 8 in 50.3% of patients (average 3) and ≥ 8 (average 14) in 49.7% of patients in this cohort [17]. Mean age is 66 years, with an average prostate volume from 49 to 56 mL [17]. Data on *Q*_max_ and PVR and prostate volume are not available from our institutional rPx cohorts, as BPH‐related voiding dysfunction is not the indication for surgery in these patients.

Radical prostatectomy was performed exclusively for treatment of prostate cancer. Patients with prior surgery for BPH were excluded from tissue collection in this cohort. Tissues were sampled from the periurethral zone by a pathologist within 1 h after surgical removal of the prostate, following longitudinal opening of the prostate from the capsule to the urethra and visual assessment of the intersections for tumor infiltration. Prostates with macroscopically tumor infiltration in the periurethral region were rare (< 1% of prostates), consistent with the characteristic predominance of tumors in the peripheral zone [18, 19], but were excluded from sampling. HoLEP and ThuLEP were carried out using a three‐lobe enucleation approach, and morcellated tissue fragments were collected and used in organ bath experiments as recently described [20, 21]. For intermediate storage and transport, organs and tissues were kept in Custodiol solution, with the prostate specimens being placed in containers containing Custodiol immediately after removal in the operating room and after sampling by pathologists. Organ bath experiments were initiated within 3 h following surgical removal of the prostate or tissue extraction from patients. This study was carried out in accordance with the Declaration of Helsinki of the World Medical Association and has been approved by the ethics committee of Ludwig Maximilian University, Munich, Germany. Written informed consent was obtained from all patients. All samples were collected and analyzed anonymously. All surgeries were performed at the Department of Urology of the LMU University Hospital.

### 2.2 Organ Bath

Tissue strips measuring approximately 6 × 3 × 3 mm were obtained from rPx tissues by cutting, while comparably sized samples from laser‐enucleated tissues were selected from the largest available shreds without additional cutting. Samples were mounted in four‐channel myograph systems (Model 720 M; Danish Myotechnology, Aarhus, Denmark) containing 10 mL Krebs–Henseleit solution at 37°C, continuously gassed with carbogen (95% O_2_, 5% CO_2_). Tissues were equilibrated to a stable resting tension of 4.9 mN over 45 min, and subsequently contracted by stimulation with 80 mM KCl. Following attainment of the maximal contraction plateau, chambers were washed to restore baseline tone. Subsequently, inhibitors at the indicated concentrations were applied to two chambers, while the remaining two chambers received vehicle (dimethyl sulfoxide, DMSO). For each experiment, all four chambers contained tissues originating from the same prostate, allowing paired comparison of inhibitor‐treated and control samples. Thus, both groups in each experiment were determined with double determinations. After an incubation period of 30 min, concentration response curves to agonists or frequency response curves to electric field stimulation (EFS) were generated. EFS experiments were conducted as described previously [22], with only one curve obtained from each tissue strip. A 30‐min preincubation period was chosen to allow acute inhibition of Rab GTPase activity before agonist exposure. Inhibitors remained present throughout generation of concentration or frequency response curves, allowing potential effects on α_1_‐adrenoceptor trafficking following agonist‐induced internalization while minimizing longer‐term cellular adaptations. Assignment of tissues to control or inhibitor conditions was alternated between experiments.

Maximum tensions with agonists and EFS were normalized to the reference contraction induced by 80 mM KCl, to compensate for differences in strip dimensions, smooth muscle content, or any other heterogeneity. BPH is a histologically heterogeneous condition and may comprise phenotypes characterized by predominantly stromal or glandular hyperplasia, mixed patterns, and varying degrees of fibrosis, with considerable overlap between these features [23]. This phenotypic heterogeneity is reflected by substantial interindividual variation in molecular marker expression, as recently documented [15, 24, 25], but remains poorly understood. Such heterogeneity may also result in differences in contractile responsiveness between independent experimental series. To account for tissue heterogeneity and enable meaningful assessment of inhibitor effects, contractions were normalized to contractions induced by high‐molar KCl, and a paired experimental design was used, with control and inhibitor‐treated samples obtained from the same prostate. This approach allows the detection of drug effects, despite histological and clinical variation [20].

*E*_max_ values for agonists and EFS, and EC_50_ values for agonists and EF_50_ values for EFS (frequency inducing half‐maximal EFS contraction), were determined separately for each individual experiment by non‐linear curve fitting [26], based on the means of double determinations and using GraphPad Prism 6 (GraphPad Software Inc., San Diego, CA, USA). The program shows error messages if curve fitting fails, or marks the results as “ambiguous” if they appear ambiguous or implausible. As recommended by the manufacturer (“GraphPad Curve Fitting Guide,” GraphPad Software Inc.), results from curve fitting were also manually checked for plausibility. Error messages or implausible results occur most frequently if the concentration response curves show pronounced “downhill” parts at higher agonist concentrations. Consequently, all curves were manually checked, and parts beyond concentrations showing the maximum contraction were excluded from curve fitting analysis if curve fitting was problematic due to sustained downhill parts. However, downhill parts were not excluded for diagrams or for analyses of concentration response curves. For some concentration response curves, maximal contraction was apparently not reached within the applied agonist concentration ranges, resulting in non‐sigmoidal curve shapes. In these cases, values were derived directly from the experimental curves, that is, the highest contractions obtained at 100 μM were used as *E*_max_ values, while 100 μM was assumed as EC_50_. In scatter plot diagrams, these values were indicated in gray. In a few cases, individual values were obviously implausible, but could be plausibly estimated from the corresponding curves. These values were likewise indicated in gray. In addition to curve fitting, area under the curves (AUC) were calculated individually for each single experiment, using GraphPad Prism 6.

### 2.3 Drugs and Nomenclature

CID1067700 (2‐(benzoylcarbamothioylamino)‐5,5‐dimethyl‐4,7‐dihydrothieno[2,3‐c]pyran‐3‐carboxylic acid) is a Rab GTPase inhibitor reported to display highest affinity for Rab7 (*K*_i_ 13 nM) [27]. Nexinhib20 ((Z)‐4,4‐dimethyl‐1‐(3‐nitrophenyl)‐2‐(1,2,4‐triazol‐1‐yl)pent‐1‐en‐3‐one) is a Rab27 inhibitor, inhibiting Rab27‐mediated exocytosis with an IC_50_ of 330 nM [28]. Stock solutions (1000‐fold of final concentrations) were prepared in DMSO and stored as small aliquots at −20°C until use. Phenylephrine ((R)‐3‐[‐1‐hydroxy‐2‐(methylamino)ethyl]phenol) and methoxamine (α‐(1‐Aminoethyl)‐2,5‐dimethoxybenzyl alcohol) are α_1_‐selective adrenoceptor agonists. Stock solutions (10 mM) of noradrenaline, phenylephrine and methoxamine were freshly prepared in deionized water before each experiment. Noradrenaline, phenylephrine and methoxamine were obtained from Sigma‐Aldrich (St. Louis, MO, Germany), CID1067700 from MedChemExpress (Monmouth Junction, NJ, USA) and nexinhib20 from Bio‐Techne (Minneapolis, MN, USA).

### 2.4 Data and Statistical Analyses

Data are presented as means ± standard deviations in concentration and frequency response curves, and as individual values from each experiment together with means in scatter plots for results from curve fitting. In the text, data are reported as means ±95% confidence intervals (CIs), and as mean differences (MD) with 95% CI. As tissues used for control and inhibitor groups originated from the same prostate in each experiment, data were analyzed using paired statistics. Concentration and frequency response curves were compared by two‐way ANOVA, for which no non‐parametric alternative is available. For data from curve fitting, parametric and non‐parametric tests were applied as indicated in the legends to figures and pending on normality. Normality was assessed by the Shapiro–Wilk normality test. Comparisons were performed by Student's *t*‐tests if data were normally distributed in both groups, or by Wilcoxon matched‐pairs signed rank or Mann Whitney test if data showed no normal distribution in at least one of both groups. Data analyses were performed using GrapPad Prism 6 (GraphPad Software Inc., San Diego, CA, USA), except for normality tests, which were performed using GraphPad Prism 9.4. *p* values are shown only if < 0.05. No predefined group sizes were specified, but a sample size of *n* = 5 independent experiments per series was generally intended. In two series, additional experiments were performed following interim evaluation of five experiments and re‐assessment of experimental datasets during the course of the study. Consequently, and because the study was not based on a strict, pre‐specified study protocol, the experiments should be regarded as exploratory in nature, and *p* values are therefore interpreted descriptively. Interpretations and conclusions are based on effect sizes and their potential biological relevance, rather than *p* values.

## 3 Results

### 3.1 Effects of CID1067700 on Laser‐Enucleated Prostate Tissues

Phenylephrine, methoxamine, noradrenaline and EFS induced concentration‐ or frequency‐dependent contractions of laser‐enucleated tissues, which were not consistently changed by 1 μM (Figure 1a–d) or 10 μM (Figure 1e–h) CID1067700. A significant decrease of noradrenaline‐induced contractions at 1 μM CID1067700 observed in concentration response curves was paralleled by unchanged AUCs and *E*_max_ values (Figure 1c). Phenylephrine‐, methoxamine‐ and EFS‐induced contractions were similar in controls and with 1 μM CID1067700 (Figure 1a,b,d). A significant increase of phenylephrine‐induced contractions at 10 μM CID1067700 observed in concentration response curves was paralleled by increased AUCs and *E*_max_ values (Figure 1e). However, methoxamine‐, noradrenaline‐ and EFS‐induced contractions were similar in controls and with 10 μM CID1067700 (Figure 1f–h). EC_50_ values for agonists, and EF_50_ values for EFS were unchanged at both concentrations (Figure 1).

**FIGURE 1:**
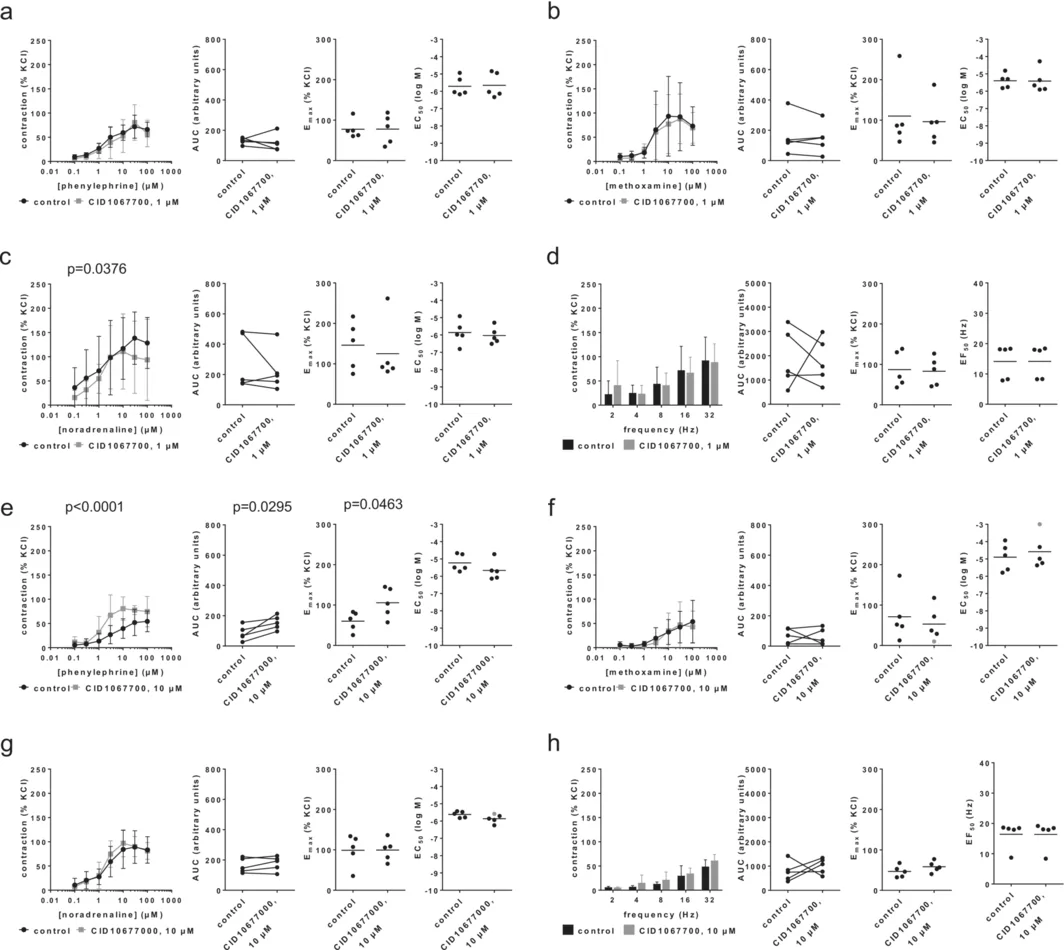
Effects of CID1067700 on contraction of human prostate tissues from laser enucleation for BPH. Concentration response curves for α_1_‐adrenergic agonists and frequency response curves for EFS were recorded 30 min after addition of 1 μM (a–d) or 10 μM CID1067700 (e–h), and solvent (DMSO) to controls. Data are from five independent experiments per panel, in which tissue samples from each patient were split to control and CID1067700‐treated groups. Data are means ± standard deviation, and individual values from each experiment, shown together with *p* values from two‐way ANOVA in concentration response curves and from Student's *t*‐test in scatter plots, if < 0.05. Values marked in gray in scatter plots reflect the maximum responses in curves (for *E*_max_) and the highest applied concentration (instead of EC_50_), as curve fitting did not provide plausible results for these single curves.

### 3.2 Effects of CID1067700 on Prostate Tissues Without Prior BPH Surgery

Phenylephrine, methoxamine, noradrenaline, and EFS‐induced concentration‐ or frequency‐dependent contractions of prostatectomized tissues without prior BPH surgery were not consistently changed by 1 μM (Figure 2a–d) or 10 μM (Figure 2e–h) CID1067700. A significant decrease of phenylephrine‐induced contractions at 10 μM CID1067700 observed in concentration response curves was paralleled by unchanged AUCs and *E*_max_ values (Figure 2e). However, methoxamine‐, noradrenaline‐, and EFS‐induced contractions were similar in controls and with 10 μM CID1067700 (Figure 2f–h). EC_50_ values for agonists and EF_50_ values for EFS were unchanged at both concentrations (Figure 2).

**FIGURE 2:**
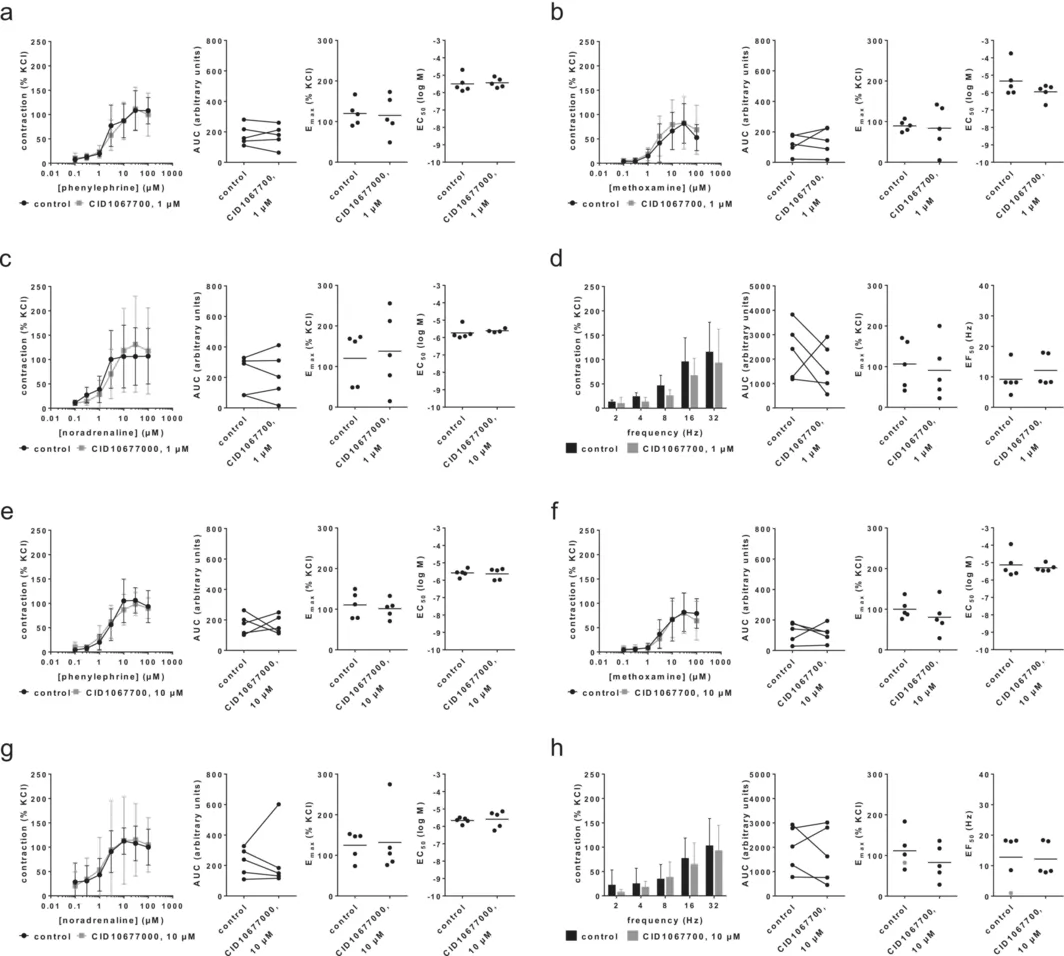
Effects of CID1067700 on contraction of human prostate tissues without prior BPH surgery. Concentration response curves for α_1_‐adrenergic agonists and frequency response curves for EFS were recorded 30 min after addition of 1 μM (a–d) or 10 μM CID1067700 (e–h), and solvent (DMSO) to controls. Data are from five independent experiments per panel, in which tissue samples from each patient were split to control and CID1067700‐treated groups. Data are in concentration and frequency response curves are means ± standard deviation. Values marked in gray in scatter plots reflect the maximum response in the corresponding curve (for *E*_max_) and an EF_50_ directly estimated from the curve, as the curve could not be converged by curve fitting.

### 3.3 Effects of Nexinhib20 on Laser‐Enucleated Prostate Tissues

Agonist‐induced contractions of laser‐enucleated tissues were not changed by 1 μM (Figure 3a–d) or 10 μM (Figure 3e–h) nexinhib20. At 1 μM nexinhib20, EFS‐induced contractions were unchanged (Figure 3d). At 10 μM, EFS‐induced contractions were reduced by nexinhib20, reflected by decreased contractions in frequency response curves, decreased AUCs and decreased *E*_max_ values (Figure 3h). The *E*_max_ reduced from 79% [34–124] of KCl‐induced contractions in controls to 40% [6–74] of KCl‐induced contractions with 10 μM nexinhib20 (MD −39% [−69 to −9] of KCl). The AUC (arbitrary units) reduced from 1562 [550–2574] in controls to 744 [17–1472] with 10 μM nexinhib20 (MD −817 [−1532 to −103]). EC_50_ values for agonists and EF_50_ values for EFS were unchanged at both concentrations (Figure 3).

**FIGURE 3:**
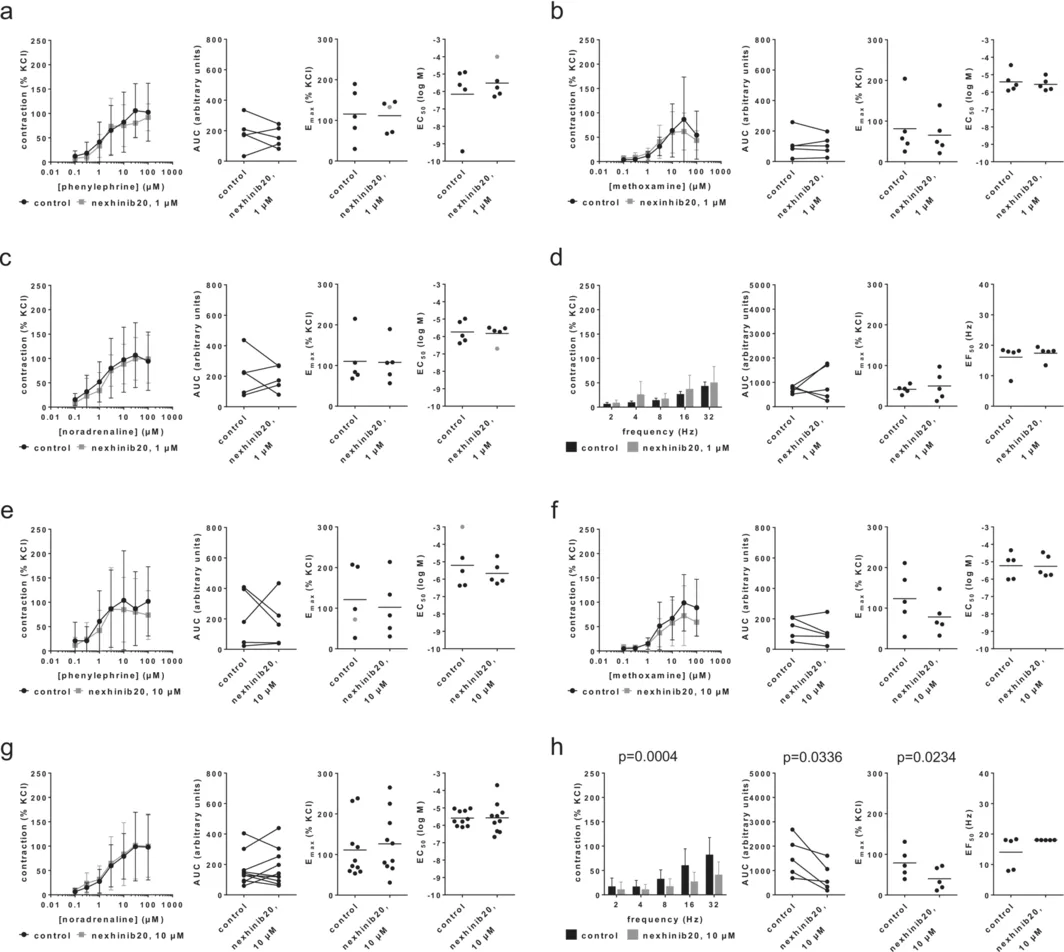
Effects of nexinhib20 on contraction of human prostate tissues from laser enucleation for BPH. Concentration response curves for α_1_‐adrenergic agonists and frequency response curves for EFS were recorded 30 min after addition of 1 μM (a–d) or 10 μM nexinhib20 (e–h), and solvent (DMSO) to controls. Data are from five to 10 independent experiments per panel (*n* = 10 for noradrenaline with 10 μM, *n* = 5 for all others), in which tissue samples from each patient were split to control and nexinhib20‐treated groups. Data are means ± standard deviation, shown together with *p* value from two‐way ANOVA in frequency response curves and from Student's *t*‐test in scatter plots, if < 0.05. Values marked in gray in scatter plots were estimated directly from concentration response curves (noradrenaline with 1 μM), or reflect the maximum responses in curves (for *E*_max_) and the highest applied concentration (instead of EC_50_) (phenylephrine with 1 and 10 μM), as curve fitting did not provide plausible results for these single curves.

### 3.4 Effects of Nexinhib20 on Prostate Tissues Without Prior BPH Surgery

Agonist‐ and EFS‐induced contractions of prostatectomized tissues without prior BPH surgery were not consistently changed by 1 μM (Figure 4a–d) or 10 μM (Figure 4e–h) nexinhib20. At 1 μM, a significant increase in phenylephrine‐induced contractions observed in concentration response curves (Figure 4a) was paralleled by unchanged methoxamine‐, noradrenaline‐, and EFS‐induced contractions (Figure 4b–d). At 10 μM, significant decreases in phenylephrine‐induced contractions (Figure 4e) and significant increases in methoxamine‐induced contractions (Figure 4f) were paralleled by unchanged noradrenaline‐ and EFS‐induced contractions (Figure 4g,h). EC_50_ values for agonists and EF_50_ values for EFS were unchanged at both concentrations (Figure 4).

**FIGURE 4:**
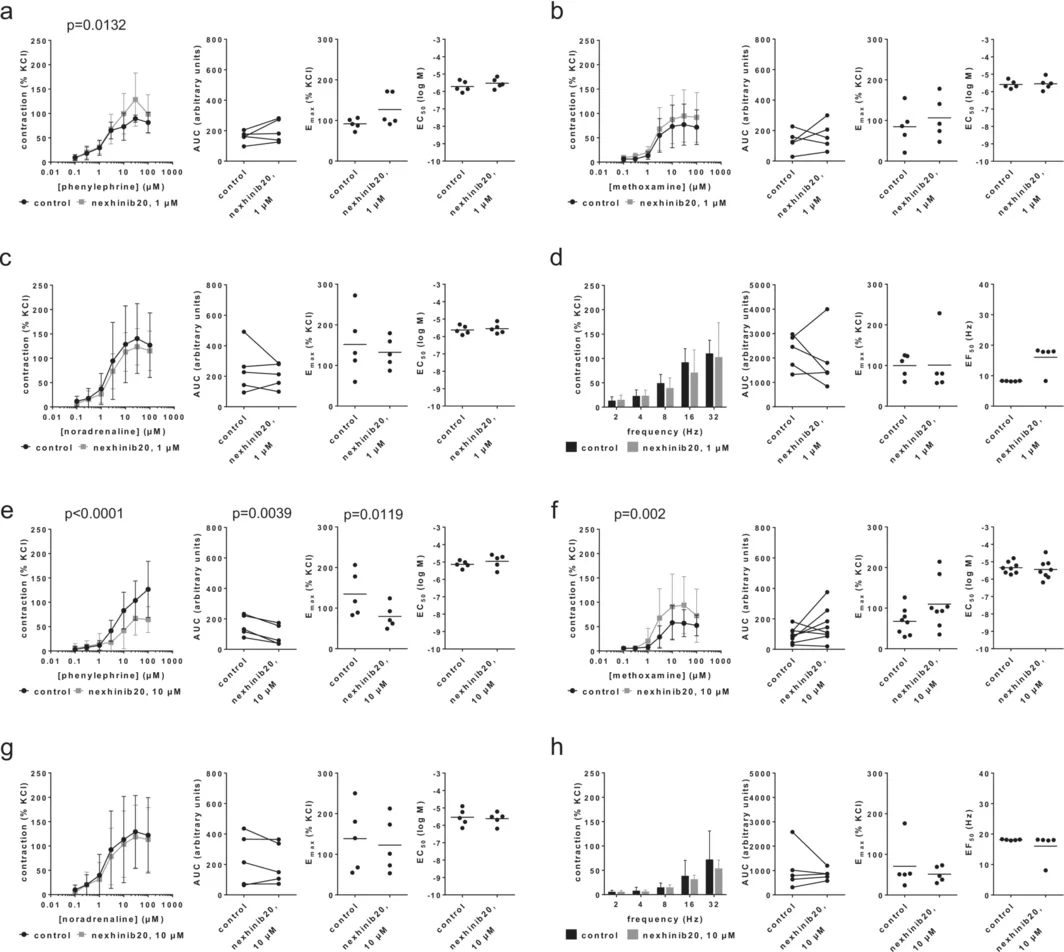
Effects of nexinhib20 on contraction of human prostate tissues without prior BPH surgery. Concentration response curves for α_1_‐adrenergic agonists and frequency response curves for EFS were recorded 30 min after addition of 1 μM (a–d) or 10 μM nexinhib20 (e–h), and solvent (DMSO) to controls. Data are from five to eight independent experiments per panel (*n* = 8 for methoxamine with 10 μM, *n* = 5 for all others), in which tissue samples from each patient were split to control and nexinhib20‐treated groups. Data are means ± standard deviation, shown together with *p* values from two‐way ANOVA in concentration response curves and from Student's *t*‐test in scatter plots, if < 0.05.

## 4 Discussion

Our findings emphasize that regulatory mechanisms identified in cell culture models with receptor overexpression and in biochemical assays may not necessarily translate to endogenous receptor function in human tissues. Previous evidence demonstrated Rab7‐mediated trafficking of internalized α_1A_‐adrenoceptors in cell culture models with receptor overexpression [7]. Noradrenaline is the endogenous neurotransmitter mediating α_1A_‐adrenoceptor‐induced smooth muscle contraction in the prostate [2], and exocytotic processes such as neurotransmission may also involve Rab GTPases including Rab27 [10]. We therefore speculated that small molecule inhibitors targeting Rab7 and Rab27 may modulate adrenergic contractions in human prostate tissues. The lack of consistent effects of CID1067700 despite previously reported Rab7‐dependent regulation of α_1A_‐adrenoceptors questions the transferability of findings from receptor overexpression systems and biochemical small GTPase assays to endogenous receptor regulation in human tissues. Divergent responsiveness of neurogenic and agonist‐induced contractions to nexinhib20 suggests a possible role of Rab27 in neurotransmission of human prostate smooth muscle.

Due to the spherical 3D structure, small size and high substrate affinity of monomeric GTPases, the development of small molecule inhibitors has been notoriously difficult [29]. CID1067700 was the first compound showing proven inhibition of a Rab GTPase [27]. In bead‐based flow cytometry assays using recombinant GSH‐Rab7, CID1067700 inhibited binding of fluorescently linked GTP (BODIPY‐GTP) to Rab7 with a *K*_i_ value of 12.89 nM, and with a maximum inhibition (≥ 97%) at 1 μM [27]. Equilibrium nucleotide binding assays indicated a competitive mechanism of inhibition, and that the inhibition was highest at low nucleotide concentrations [27]. However, its specificity appears limited. In a comprehensive follow‐up, CID1067700 inhibited BIODIPY‐GTP binding to Rab7, H‐Ras, Cdc42, RhoA and Rac1 with similar EC_50_ values in nanomolar ranges [29]. Inhibitions became maximum at 10 μM CID1067700 and were already close to the *E*_max_ at 1 μM [29]. Although the *E*_max_ was highest for Rab7, maximum inhibition still mounted to 75% for RhoA, and around 50% for Cdc42 and Rac1 [29].

RhoA is essential in smooth muscle contraction, including the prostate [30, 31, 32]. Typically, inhibition of its effector, Rho kinase, results in complete loss of contractility in smooth muscle preparations [33, 34]. In addition to RhoA, contraction of prostate smooth muscle cells may be driven by Cdc42 and Rac1, since Rac1 inhibitors inhibit contractions of human prostate tissue [25, 35], and Cdc42 silencing inhibits contractions of stromal cells [36]. Given the lack of effects of 1–10 μM CID1067700 in our experiments, but inhibitions of 50%–75% for RhoA, Cdc42, and Rac1 in biochemical assays, the transferability of data from BIODIPY‐GTP assays with recombinant GTPases to physiological settings with human tissues is obviously limited. Changes in nucleotide fluorescence due to distant allosteric interactions, in addition to or even without replacement of the fluorescence‐labeled GTP by the test compounds cannot be ruled out in BODIPY‐GTP assays [29]. In fact, the potency of CID1067700 differed markedly when it was determined by pulldown assays. Pulldown of GTP‐Rab7 by GST‐RILP beads (reflecting active Rab7) was inhibited at 80 and 120 μM CID1067700, but only weakly and not significantly at 40 μM in HepG2 cells [37]. Again, in RILP pulldown assays, CID1067700 inhibited Rab7 activity in mouse B cells at 40 μM, which was nearly complete and did not include further concentrations [38]. An in silico docking analysis revealed a secondary binding site of CID1067700, outside the GTP binding pocket [38], so that findings from fluorescence‐based assays may have been indeed affected by artifacts from allosteric binding.

Accordingly, effective concentrations for CID1067700 varied greatly in different experimental models. Endocytosis of cell surface proteins in HeLA cells was inhibited by 20 nM, which has been explained by Rab7 inhibition [39]. In contrast, 20 μM was the lowest effective concentration in human B lymphoma cells, where growth in MTS assays was inhibited with IC_50_ values from 53 to 103 μM [40]. Further observations include protection against cell injury in rat astrocytes by 80 nM CID1067700 [41], inhibition of lipid droplet–phagosome association in macrophages at 200 nM [42], alterations in morphology and key signaling pathways in differentiating adipose‐derived stem cells to keratinocytes at 40 μM [43], inhibition of β‐coronavirus egress from lysosomes at 40 μM (but not 4 μM) [44], and Rab18‐mediated vesicle secretion from human mesenchymal stem cells again at 40 μM [45].

Nexinhib20 has been developed as an inhibitor for Rab27a‐JFC1‐mediated exocytosis of azurophilic granules by neutrophils, without affecting phagocytosis or neutrophic extracellular trap production [28]. In screening of a compound library by time‐resolved Förster resonance energy transfer (TR‐FRET) assays, 10 μM reduced the interaction of Rab27a with its effector, JFC1, by approximately 50% [28, 46]. Myeloperoxidase secretion by neutrophils was inhibited with an IC_50_ of 330 nM [28]. The maximum possible inhibition of the Rab‐JFC1 interaction by nexinhib20 appears to be 50%, as the interaction was inhibited by 50% in pulldown assays using 50 μM, whereas G‐LISA assays determined an IC_50_ of 2.6 μM for the inhibition of this interaction [28]. Docking simulations suggested binding of nexinhib20 to a druggable pocket in Rab27A resulting in disruption of the effector binding, rather than a competitive mechanism of inhibition [28]. In TR‐FRET assays, nexinhib20 inhibited neither the interactions of Rab11 with Munc13‐4 at 10 or 50 μM [28], nor the interaction of Rac1 with PAK1 at 10 μM [46]. However, in pulldown assays based on PAK1 interaction, 10 μM nexinhib20 completely inhibited interleukin‐8‐ and N‐formyl‐methionyl‐leucyl‐phenylalanine‐induced Rac1 activation in HL60‐CXCR2 cells, HL60 cells, and human neutrophils, and inhibited recombinant Rac1 in vitro with an IC_50_ of 29.3 μM [47]. In HL‐60 cells, 100–500 nM nexinhib20 was not toxic, but concentration‐dependently inhibited their infection with an intracellular bacterium [48]. In three lines of small cell lung cancer cells, nexinhib20 reduced the viability with IC_50_ values of 1.55–12.19 μM and caused substantial decreases in total exosome release at these IC_50_ concentrations [49]. In mouse neutrophils, a single concentration of 10 μM was tested, which reduced the azurophilic granule exocytosis [50].

Although nexinhib20‐sensitive neutrophil exocytosis is unlikely to represent a major mechanism underlying chronic low‐grade inflammation in the prostate, chronic inflammation itself is increasingly considered an important factor driving BPH [51, 52]. Age‐related cellular senescence and the resulting senescence‐associated secretory phenotype may represent an overarching mechanism promoting chronic inflammation, which may be further aggravated by metabolic and other proinflammatory factors [51, 52, 53]. Persistent inflammation may in turn promote fibrosis, potentially reducing tissue and urethral compliance and thereby contributing to voiding symptoms [53, 54]. Interestingly, Rab7 and Rab27 have also been implicated in cellular senescence and fibrotic processes in other tissues [55, 56, 57, 58, 59], suggesting that their potential relevance to BPH may extend beyond α_1_‐adrenoceptor trafficking and neurotransmission. Such mechanisms may be particularly relevant as BPH‐related obstruction is not exclusively determined by smooth muscle contraction, and structural components such as fibrosis and reduced tissue compliance may increasingly contribute to medication‐refractory disease. Whether CID1067700 or nexinhib20 can modulate these processes in prostate tissues remains unknown. Accordingly, anti‐inflammatory strategies have been investigated for BPH, including cyclooxygenase inhibitors, but their long‐term use is limited by cardiovascular safety concerns [51]. Other approaches, including phytotherapeutics and modulation of inflammatory signaling pathways such as mTOR, are limited by uncertain efficacy, potential systemic effects or immunosuppression, and high costs.

The findings summarized above regarding *K*_i_ and IC_50_ values from previous studies are not without contradictions. However, the concentrations we used should produce partial, if not complete, inhibition of the specific targets (i.e., Rab7 and Rab27a), and may also cover the known off‐targets, including RhoA. Our results suggest that the posttranslational regulation of α_1_‐adrenoceptors in the human prostate does not appear to depend reproducibly on pathways sensitive to CID1067700 and nexinhib20. This seems to be stable across different BPH stages, as both tissue types used here showed no differences in experiments with CID1067700 and nexinhib20. Typically, patients needing laser enucleation of the prostate are characterized by advanced, medication‐refractory voiding symptoms, high PVR or complications, while the majority of patients undergoing rPx but without prior surgery for BPH show only mild or moderate symptoms [11]. Resistance of prostatic α_1_‐adrenoceptors to Rab GTPase‐mediated degradation may prevent loss of receptor function, and may potentially amplify prostate smooth muscle tone in urethral obstruction of surgical and non‐surgical BPH. At the same time, our results were characterized by pronounced variability. In some series, agonist‐induced contractions appeared changed in the presence of CID1067700 or nexinhib20. However, these effects were never consistent across all agonists tested. This variability may reflect marked heterogeneity of human prostate tissues, potentially including interindividual differences in receptor regulation, resulting in responder/non‐responder constellations regarding CID1067700 and nexinhib20. Independently from such heterogeneity, the lack of reproducible inhibitory effects under functionally relevant ex vivo conditions highlights important limitations in translating findings from receptor overexpression systems, biochemical assays, and FRET‐based small GTPase assays to endogenous receptor regulation in intact human tissues.

To obtain robust data, we repeated the agonist experiments using three different α_1_‐adrenoceptor agonists. The results were consistent insofar as no uniform pattern was observed, such as consistent increases or decreases across all three agonists (phenylephrine, noradrenaline, and methoxamine). However, under some conditions, responses differed between agonists despite identical inhibitor treatment. Therefore, the observed inhibition of EFS‐induced contractions by 10 μM nexinhib20 in laser‐enucleated tissues should be interpreted with caution despite statistical significance, as it may also reflect experimental variability or a chance finding, instead of a consistent inhibitory effect. On the other hand, the effect may reflect an influence on contractile neurotransmission. The observed pattern would be compatible with a presynaptic effect on neurotransmitter release rather than altered postsynaptic α_1_‐adrenoceptor signaling. Neurotransmitter release from synaptic vesicles represents an exocytotic process that may involve Rab GTPases and could therefore be susceptible to nexinhib20. If this represents a true inhibitory effect rather than an artifact, it appears to be restricted to laser‐enucleated BPH tissues and absent in prostatectomy specimens with only mild BPH. Although laser‐enucleated tissues have only recently been introduced as a model to study prostate smooth muscle contraction [20, 21], differences in EFS‐induced responses between these tissue types have already been reported [11]. Therefore, further studies are required to clarify the nature of the effects of nexinhib20 and to better understand the differences in neurogenic contractions between laser‐enucleated and prostatectomized tissues, including whether these arise from surgical procedures and tissue processing or reflect intrinsic differences in tissue composition.

Posttranslational regulation has been most frequently studied for β‐adrenoceptors, but rarely for α_1_‐adrenoceptors, while α_1A_‐adrenoceptors account for prostate smooth muscle contraction [2]. Outcomes of Rab‐mediated receptor internalization may vary with receptor types and ligands. Accordingly, cross‐validation using three different α_1_‐adrenoceptor agonists in our experiments reduces the possibility that the lack of consistent inhibitor effects resulted from agonist‐specific differences in receptor trafficking. In general, Rab5 mediates receptor transport from the plasma membrane to early endosomes, Rab4 the recycling of internalized receptors from endosomes back to the cell surface, Rab7 the transport from early to late endosomes, and from there to lysosomes, Rab9 from late endosomes to the Golgi, and Rab11 from early to recycling endosomes and from there to the Golgi or back to the plasma membrane [8]. In HEK 293 cells stably expressing α_1A_‐adrenoceptors, noradrenaline, methoxamine and oxymetazoline caused internalization of α_1A_‐adrenoceptors [7]. Internalization by noradrenaline and methoxamine was paralleled by interaction of α_1A_ with Rab5 and Rab7, while oxymetazoline caused interaction with Rab5 and Rab9, but not with Rab7 [7]. As Rab7 is associated with transport to lysosomes and proteasomes, it was concluded that noradrenaline‐ and methoxamine‐induced internalization finally resulted in receptor degradation, and oxymetazoline‐induced internalization in slow recycling [7]. Apart from ligand‐specific trafficking, regulation depends on the equipment of cells with different Rab isoforms. Thus, in rat cardiac myocytes, overexpression of Rab1 increased the population of α_1A_‐adrenoceptors at the cell membrane, by transport of either newly synthesized or internalized receptors from intracellular compartments to the cell surface [60]. Unlike α_1A_‐adrenoceptors, stably expressed α_1B_‐adrenoceptors in HEK 293 cells associated with Rab5, Rab4, and Rab11 following noradrenaline stimulation but not with late endosome markers, such as Rab9 and Rab7, suggesting that internalized α_1B_ may be rather recycled whereas α_1A_ seems to be degraded [61, 62].

Thus, previous evidence on Rab7‐dependent α_1A_‐adrenoceptor trafficking was obtained in receptor overexpression systems, whereas our experiments addressed endogenous receptor function in intact human tissues. Despite the lack of consistent effects in the present study, involvement of other Rab GTPases in α_1_‐adrenoceptor regulation cannot be excluded, with Rab1, Rab5, and Rab9 representing potential candidates based on previous evidence on α_1_‐adrenoceptor trafficking. Likewise, other mechanisms of posttranslational α_1_‐adrenoceptor regulation merit further investigation, as these remain poorly understood in the human prostate. Such studies are currently hampered by the limited availability of suitable pharmacological tools targeting posttranslational regulators, including, for example, arrestins [6]. In addition, reported affinities of CID1067700 and nexinhib20 largely originate from biochemical or fluorescence‐based assay systems that differ fundamentally from functional tissue experiments. While heterogeneity of human prostate tissues may contribute to variability and subgroup‐specific responses cannot be excluded, the absence of reproducible inhibitor effects under ex vivo conditions raises broader questions regarding the transferability of findings from simplified preclinical models to physiologically relevant human tissues.

## 5 Conclusions

Our findings highlight important limitations in translating results from biochemical and overexpression‐based preclinical models to functional regulation in human tissues. Despite previously demonstrated Rab7‐mediated regulation of α_1A_‐adrenoceptors by Rab7 in a cell model with stable receptor overexpression and a reported nanomolar affinity of CID1067700 for Rab7, no consistent effects of CID1067700 were observed on α_1_‐adrenergic contractions of human prostate tissues, irrespective of whether tissues originated from surgery for BPH or from patients without previous BPH surgery. Findings with nexinhib20 suggest a possible presynaptic role of Rab27a in adrenergic neurotransmission in prostate tissues from surgical BPH.

## Author Contributions

**Luca Troffer:** investigation, formal analysis, writing – review and editing. **Peibin Cen:** investigation, formal analysis, writing – review and editing. **Martin Hennenberg:** conceptualization, data curation, formal analysis, investigation, methodology, project administration, resources, supervision, writing – original draft. **Michael Brandstetter:** investigation, formal analysis, writing – review and editing. **Alexander Tamalunas:** investigation, writing – review and editing. **Leo Stadelmeier:** investigation, writing – review and editing. **Philipp Weinhold:** investigation, writing – review and editing. **Patrick Keller:** investigation, writing – review and editing. **Yajie Xu:** investigation, formal analysis, writing – review and editing. **Christian G. Stief:** conceptualization, investigation, resources, supervision, writing – review and editing.

## Funding

The authors have nothing to report.

## Ethics Statement

This study was performed using human prostate tissues from radical prostatectomy for prostate cancer and from laser enucleation for benign prostatic hyperplasia. The study was carried out in accordance with the Declaration of Helsinki of the World Medical Association and has been approved by the ethics committee of the Ludwig‐Maximilians University, Munich, Germany (approval numbers 22‐0827, 22‐0608).

## Consent

Written informed consent was obtained from all patients.

## Conflicts of Interest

The authors declare no conflicts of interest.

## Data Availability

All data generated or analyzed during this study are included in this published article. Original and raw data are available from the corresponding author on reasonable request.
